# Something Big that Matters: The American Society of Tropical Medicine and Hygiene’s Commitment to Combat Climate Change

**DOI:** 10.4269/ajtmh.20-1267

**Published:** 2020-10-09

**Authors:** Chandy C. John

**Affiliations:** Ryan White Center for Pediatric Infectious Disease and Global Health, Indianapolis, Indiana

Climate change is the most pressing global health issue of our time. Climate change has had and will have profound effects on human health, including the health issues most important to the members of the American Society of Tropical Medicine and Hygiene (ASTMH). As the leading global health society, the ASTMH is dedicated to combating climate change and improving planetary health. Attention to climate change is part of the ASTMH 2019–2022 strategic plan, and climate change and planetary health will be key components of future strategic plans. But, what about ASTMH’s contribution to climate change? In the current issue of the *American Journal of Tropical Medicine and Hygiene*, Teun Bousema and fellow ASTMH members conducted a superb and insightful analysis on the carbon footprint of the ASTMH 2019 Annual Meeting in National Harbor, Maryland. The findings are revelatory. The meeting is a carbon emission hog: 27.7 million miles traveled, and almost 8,700 metric tons of carbon dioxide equivalent emissions, equal to the weekly carbon footprint of about 9,400 American households. What can we do to reduce these carbon emissions?

In many ways, the carbon footprint of the ASTMH Annual Meeting indicates the international nature of this meeting. Although we are named the ASTMH, we are an international society. A substantial portion of the carbon footprint comes from participants from distant countries, many of them low- and middle-income countries (LMICs), flying to attend the meeting in the United States. This level of international travel is greater than that for most other “American” society meetings. The strong representation of members and others from LMICs in which diseases such as malaria, helminth infections, and arthropod-borne viral infections are endemic is one of the greatest strengths of the ASTMH Annual Meeting. At the meeting, there is no substitute for physical proximity, to facilitate discussion and networking, and allowing participants a personal sense of the world of tropical medicine and global health. How can we meet that goal while decreasing the huge carbon cost of the meeting? Bousema et al.^1^ propose a number of potential steps in the right direction, including alternating between a physical and virtual meeting, offering a hybrid in-person/online conference, or decentralizing the meeting, with multiple simultaneous conference venues. Each of these options would result in substantial carbon emission savings and is worth taking seriously. But, each also has down sides, notably the loss of in-person interaction and increased logistical challenges.

Enter COVID-19. This ubiquitous coronavirus and the pandemic it created have led to a real-life experiment. The 2020 ASTMH Annual Meeting will be a virtual conference, given the difficulty of safe travel during the current phase of the pandemic. The 2020 meeting will reduce our carbon footprint to almost zero, one tiny silver lining to the cloud of COVID-19. It will also begin to teach us what works and does not work virtually, and help us to set up future meetings that build on this knowledge. Hybrid meetings that combine in-person and virtual meetings for different attendees are likely to follow. This format may offer a simple next step that decreases carbon footprint while increasing overall participation. If well managed, a hybrid format may offer an unprecedented opportunity to allow many more interested participants, particularly from LMICs, to attend the ASTMH Annual Meeting. But, the hybrid format will require thoughtful implementation to ensure that it does not result in a “two-tier” conference, in which those who cannot afford to pay for travel experience a less immersive and connected experience than those who can afford to attend in person. Some of the same concerns come up when considering a plan for multiple simultaneous conference venues. Short of a multisite format for the Annual Meeting, partnering with different organizations for an ASTMH presence at regional meetings, as has taken place in recent years at conferences in Asia, Africa, and South America, may be a useful step toward the goal of enhancing regional participation for those unable to attend the Annual Meeting in North America.

The ASTMH must also commit to planetary health beyond reduction in carbon emissions. We must build on our strengths in advocacy to push, in partnership with like-minded groups, for policies that combat climate change, particularly those that relate directly to global health. We must advocate for conservation of water, a vital resource strongly linked to population health. Preservation of the environment, including reduction of pollutants and preservation of lands that are needed for other species to survive, is inextricably linked to human health. A prime example of this interconnectedness is the transmission of SARS-CoV-2, hantaviruses, and other pathogens from animals to humans, when there is increased contact as humans encroach on the animals’ territories. These are not areas in which the ASTMH has traditionally worked or spoken up, but in fact environmental conditions may influence the diseases we study more than many other factors we currently assess. Partnership with groups who are expert in these areas, consideration of how the ASTMH can facilitate our understanding of how environmental problems affect diseases endemic to LMICs, and working to effect needed change are all areas the ASTMH should explore in efforts to improve health for underserved populations globally.

When we do return to an in-person or hybrid in-person/virtual meeting, the ASTMH, led by the “Green Working Group,” should work with meeting venues to ensure better environmental practice—less waste, more recycling, and responsible use of energy resources at the venue. And, of course, the ASTMH as an organization should promote personal measures by members to reduce carbon emissions and waste, encouraging reduced consumption in general, responsible water use, recycling, and the use of public transport once we are past COVID-19 strictures. These measures may seem out of the society’s mandate at first glance, but they relate closely to our overall goal of improved global health, and take our commitment beyond the meeting into the broader world of our members.

In my presidential address in 2019, I focused on the importance of doing “something small that matters”: how the small everyday things we do can make a difference in global health. Working to combat climate change is a complementary task: “something big that matters.” Doing our society business and conducting our Annual Meeting in a year of no travel will teach us a lot. The ASTMH must build on those lessons to make concrete decisions that will reduce our carbon, waste, and water footprints. The coming years offer a unique opportunity to establish the ASTMH as a leader in improving the health of not only those people we serve but also the source that sustains us all, our planet.
